# Development of green micellar HPLC–DAD method for simultaneous determination of some sulbactam combinations used in COVID-19 regimen

**DOI:** 10.1186/s13065-023-01006-0

**Published:** 2023-08-05

**Authors:** Gamal H. Ragab, Hanaa M. Saleh, Nermeen M. Abdulla, Eman A. Bahgat

**Affiliations:** Pharmaceutical Analytical Chemistry Department, Faculty of Pharmacy, Zagazig University, Zagazig, 44519 Egypt

**Keywords:** HPLC–DAD, Green micellar method, Antibiotics combinations, Polyoxyethylene-23-lauryl ether (Brij-35), Sodium dodecyl sulfate (SDS)

## Abstract

In a new attempt to separate some sulbactam combinations by green chemical method we came up with this research in which an ecofriendly, green, sustainable and selective method was established for separation of four antibiotics, namely, cefoperazone (CFP), cefixime (CFX), ampicillin (AMP) and sulbactam (SLB). No organic solvents were used in the composition of the mobile phase as it was replaced by mixing two surfactants together, sodium dodecyl sulfate (SDS) and polyoxyethylene-23-lauryl ether (Brij-35). Effect of varying the concentrations of the two surfactants on chromatographic separation was studied. Optimum separation was maintained using a mobile phase consisting of 0.01 mol/L SDS, 0.03 mol/L Brij-35, 0.4% Tri-ethylamine (TEA) and pH of 2.8 adjusted by using 1 M ortho-phosphoric acid on reversed phase Isère C_18_ BDS column with temperature of 40 °C at flow rate 1 mL/min, wavelength 215 nm, and the total run time was 6 min. Validation of the proposed method has been made according International Conference of Harmonization (ICH) guidelines at linearity range of 10–200 µg mL^−1^ for all drugs under study, high accuracy results (recovery range 98.39–100.35%). and the variation coefficient (RSD) of the points on the calibration curve was ranged from (0.1–1.7%) indicating precise method. The LOQ was (6.09 μg mL^−1^) for CFP, (6.07 μg mL^−1^) for CFX, (3.85 μg mL^−1^) for AMP and (7.20 μg mL^−1^) for SLB. Successful applications were made on marketed dosage forms with recovery range of (100.16–102.25%) and RSD of (0.03–1.88%). The method was verified on the Green Analytical Procedure Index (GAPI) and Analytical Greenness metric approach (AGREE) and it was found to be an excellent green alternative method.

**Supplementary Information:**

The online version contains supplementary material available at 10.1186/s13065-023-01006-0.

## Introduction

Bacterial diseases have been easily controlled by antibiotics. Its effect looks like magic which prompted people to use it voraciously even if the case has no need, this lead to high consumption and sales of antibiotics all over the world. The development of antibiotics for therapeutic use was undoubtedly the most significant medical advance of the twentieth century. Antibiotics not only treated infectious infections but also made many modern medical operations possible, such as open-heart surgery, cancer treatment, and organ transplants [1]. The drugs under study belongs to different classes of antibiotics, AMP and SLB belong to penicillin and β lactamase inhibitors while, CFX and CFP belong to 3rd generation cephalosporins. Chemical structure of the studied drugs was shown in Additional file 1: Table S1. The studied drugs are listed in the United States [2] and British pharmacopeia [3].

Drugs under study had also a significant role in management of Coronavirus in the form of SLB/CFP combination for injection [4]. AMP and CFX also were used either alone or in combination with SLB [5]. SLB/CFP combination was used mainly in treatment of urinary tract infections [6] and was found to be effective for respiratory tract infections caused by beta-lactamase producing and non-producing bacteria [7]. AMP/SLB combination was used in treatment of aspiration pneumonia and lung abscess [8] and intra-abdominal infections of bacterial origin [9]. CFX was used in treatment of upper respiratory tract infections and otitis media [10].

From the previous data, it's clear that drugs under study used in treatment of most common diseases between peoples, so it has high global consumption. Many analytical methods were established to reach a sensitive, cost saving and ecofriendly method. Different techniques were utilized for analysis of the aforementioned drugs such as spectrophotometric, electrochemical and chromatographic methods. Chromatographic methods, notably the RP-HPLC methodology, offer an advantage over other approaches since they are a rapid, sensitive, and effective tool for separating and quantifying a variety of analyte mixtures. RP-HPLC methods, on the other hand, usually use huge amounts of organic solvents and generate considerable amounts of waste that must be disposed of, creating environmental and operator safety risks. The main goal is to reduce the impact of hazardous solvents by replacing conventional organic solvents (such as acetonitrile and methanol) with more environmentally friendly solvents.

Micellar liquid chromatography (MLC), one of the most popular techniques, uses entirely aqueous micellar mobile phases [11]. In this study, we utilize MLC as a new green method for analysis of the drugs under study. MLC is a reversed-phase liquid chromatographic mode that uses an aqueous surfactant solution above the critical micellar concentration (CMC) as the mobile phase. Because of their low cost and low environmental impact, utilizing pure micellar solutions as mobile phases is a very appealing proposal [12]. The stationary phases' characteristics are changed by the adsorbed surfactant monomers, while the mobile phase's micelles enhance the solubilizing effect on the eluents. The interactions described above have a significant impact on selectivity and retention behavior of the studied drugs. An anionic surfactant, SDS, is often employed in MLC. C_12_H_25_SO_4_ Na^+^ is its molecular structure. SDS monomers strongly adsorb positively charged analytes on the modified reversed stationary phase. Brij-35 is a nonionic surfactant that is used less frequently in MLC. C_12_H_25_ (OC_2_H_4_)_23_OH is its molecular formula. Brij-35 has the similar property of reducing polarity to change the reversed stationary phase surface characteristics. The stationary phase interacts with the non-ionic and ionic surfactants, giving hydrophobic and electrostatic sites of contact, respectively. SDS is mixed with Brij-35 to produce a mixed micellar mobile phase for drug separation without the need of organic solvents. Because the hydrophobic sites reduce the retention of polar chemicals, there is no need to use an organic solvent [13].

The selected drugs were analyzed many times by reported HPLC methods but all these methods are suffering from utilizing organic solvents in different proportions. There is only one HPLC method was reported for simultaneous separation of three of the drugs under study, AMP, CFP, and SLB using β-cyclodextrin as stationary phase [14]. AMP was analyzed using an UPLC/MS method [15] while, CFP and SLB was separated using different HPLC methods utilizing mixed aqueous and organic mobile phase solvents [16]. Also, an UPLC/MS method was published for determination of SLB and AMP [17]. CFX and SLB was also assayed using a RP-HPLC method [18]. In addition, a HPTLC method has been published for determination of CFP and SLB [19]. Also, a micellar capillary electrokinetic chromatographic method was reported for simultaneous determination of AMP and SLB [20]. All the previously reported methods are suffering from using organic solvents which have a high ecological impact.

Finally, the aim of the work is to develop a method free from organic solvents so it is ecofriendly, cost saving and time effective. Effect of variation of mobile phase components concentrations on separation was studied. Calibration plots and assay of marketed dosage forms were established. Analysis of the data led to the high response predictability optimization of critical variables, comparing the evaluated method's greenness to those found in previously published methods. The chosen analytes were analyzed successfully in their pharmaceutical formulation.

## Experimental

### Equipments

Waters Alliance 2695 HPLC instrumentation consisting of quaternary pump (Waters, USA), solvent cabinet with auto-sampler injector was used for chromatographic separation. Detection was carried out using Waters 2996 photodiode array (Waters, USA) with standard flow cell (10 mm path length, 1000 psi maximum pressure) connected to column compartment ISERA C_18_ BDS 100 × 4.6 mm, 3 um made in Germany. Data acquisition was done using empower 3 software (Waters, USA). All calculations were performed using Microsoft Excel 2013 (Microsoft Corporation, USA). Adjustment of mobile phase pH was carried out using benchtop lab pH meter model AD1030 (ADWA, Romania). Analytical balance, model SA 210D Scientech, USA was used for weighing standards, tablets and reagents. Digital hot plate stirrer, model JSHS-18A, JSR, korea. During study, auto-sampler injection volume was 20 μL, temperature of the column was kept at 40° C and detection wavelength was 215 nm.

### Materials and reagents

#### Chemicals and reagents

Analytical grades were utilized for all reagents. Brij-35 was obtained from Alfa Aesar, and SDS was acquired from Himedia (Mumbai, India) (Thermo Fisher Scientific Kandel, Germany). Merck provided sodium hydroxide, orthophosphoric acid, and methanol (Darmstadt, Germany). The source of the TEA was SDFCL (Sd Fine Chem Limited, India).

#### Pure samples

Pharmaceutical-grade antimicrobials were used. CFP and CFX for this study were provided by EIPICO Company (10th of Ramaden city, Egypt). The company in charge of delivering AMP and SLB was Pharco Pharmaceuticals Inc. (Alexandria, Egypt).

#### Pharmaceutical formulations

Three pharmaceutical preparations were provided from the market for analysis by method under study to prove it’s validation. Unictam® vials (Mup) is labeled to contain 1000 mg AMP and 500 mg SLB. Sulbacef® vials (Advocure) is labeled to contain 1000 mg CFP and 500 mg SLB. Suprax® capsules (Hikma Pharma) are labeled to contain 400 mg CFX.

### Standard solutions

For studying the influence of altering the mobile phase concentration components on the chromatographic separation of drugs under study, standard solutions were prepared by dissolving 0.1 g from each drug in 200 mL solvent mixture water: MeOH (3:1) to give concentration of 500 μg mL^−1^ for each drug then standard working solution of 100 µg mL^−1^ for all studied drugs was prepared by taking suitable volume of the stock solution and diluted by distilled water.

For the quantitative validation study the working solutions used were then prepared by serial dilutions of the stock solutions to the required concentrations using distilled water. For linearity, seven concentrations were prepared at levels of 10, 25, 50, 75, 100, 150 and 200 μg mL^−1^.

For accuracy and precision, the following laboratory mixtures were prepared: 50 μg mL^−1^ for quality control low (QC_L_); 100 μg mL^−1^ for quality control medium (QC_M_); and 150 μg mL^−1^ for quality control high (QC_H_) for all the studied drugs. Refrigeration of all prepared solutions was made at 2–8 °C.

#### For mobile phase preparation

0.01 mol/L SDS (0.288 g in 100 mL distilled water), 0.03 mol/L Brij-35 (3.597 g in 100 mL distilled water), 0.4% Tri-ethylamine (TEA) (0.4 mL in 100 mL) and pH of 2.8 adjusted by using 1 M ortho-phosphoric acid.

### Assay of marketed dosage forms

For preparation of marketed Unictam® vial dosage forms 1500 mg (1000 AMP + 500 SLB), stock solution was prepared by dissolving amount of the powder for injection equivalent to 0.15 g of pure drugs (0.1 g AMP and 0.05 g SLB) in 200 mL distilled water. The working solutions were prepared by taking 5, 10 and 15 mL of the stock solution and make dilution to 50 mL using distilled water to give concentrations of 50, 100, 150 μg mL^−1^ and 25, 50, 75 μg mL^−1^ for AMP and SLB, respectively.

For preparation of marketed Sulbacef® vial dosage forms 1500 mg (1000 CPF + 500 SLB), stock solution was prepared by dissolving amount of the powder for injection equivalent to 0.15 g of pure drugs (0.1 g CFP and 0.05 g SLB) in 200 mL distilled water. The working solutions were prepared by taking 5, 10 and 15 mL of the stock solution and make dilution to 50 mL using distilled water to give concentrations of 50, 100, 150 μg/mL and 25, 50, 75 μg mL^−1^ for CFP and SLB, respectively.

For preparation of marketed Suprax® capsule dosage form 200 mg CFX, stock solution was prepared by weighing ten capsules and dissolving amount of the powdered capsule equivalent to 0.1 g of CFX in 200 mL solvent mixture water: MeOH (3:1). The working solution was prepared by taking 5, 10 and 15 mL of stock solution and make dilution to 50 mL using distilled water to give concentrations of 50, 100 and 150 μg mL^−1^.

### Chromatographic conditions

Optimization of chromatographic conditions resulted in using mobile phase consisting of 0.01 mol/L SDS, 0.03 mol/L Brij-35 and 0.4% TEA for determining the cited drugs. pH was adjusted at 2.8 by using 1 M ortho-phosphoric acid, flow rate was 1 mL/min using isocratic elution and the temperature was kept at 40 °C.

To increase the sustainability of the process, the mobile phase was reused in between chromatographic runs. Additionally, the apparatus was frequently flushed with water, followed by a 15 min purge in a solution of water and MeOH (1:1) to remove the adsorbed surfactants from the stationary phase.

## Results and discussion

The presence of organic solvents in mobile phase composition represent a defect in analytical method as it has high impact on the environment, so minimizing amount of organic solvents will decrease the problem and its exclusion will eliminate the problem completely. The organic solvents were replaced by mixing two surfactants (SDS and Brij-35), optimization of chromatographic condition was studied to improve the chromatographic performance.

### Method optimization

#### Optimization of mobile phase

The mobile phase solutions of 0.01 mol/L SDS and 0.02 mol/L Brij-35 were used as a reference to evaluate the effects of varying the ratios of the two surfactants. SDS was added to the 0.02 mol/L Brij-35 solution at concentrations of 0.005, 0.01, 0.015, and 0.02, whereas Brij-35 was added to the 0.01 mol/L SDS solution at concentrations of 0.01, 0.02, 0.03, and 0.04 mol/L. TEA was added to 0.01 mol/L SDS and 0.03 mol/L Brij-35 at concentrations of 0.1%, 0.2%, 0.3%, and 0.4%. pH was adjusted to 2.8 by 1 M ortho-phosphoric acid.

The selection of the mobile phase's pH value took into account the component types (neutral or ionic), drug pka values, the nature of the silica beds, and the trials made at different pH values to achieve the best resolution conditions. The pH of choice was adjusted to be 2.8. Furthermore, appropriate anionic SDS and non-ionic Brij-35 were used to improve drug separation.

**Fig. 1:**
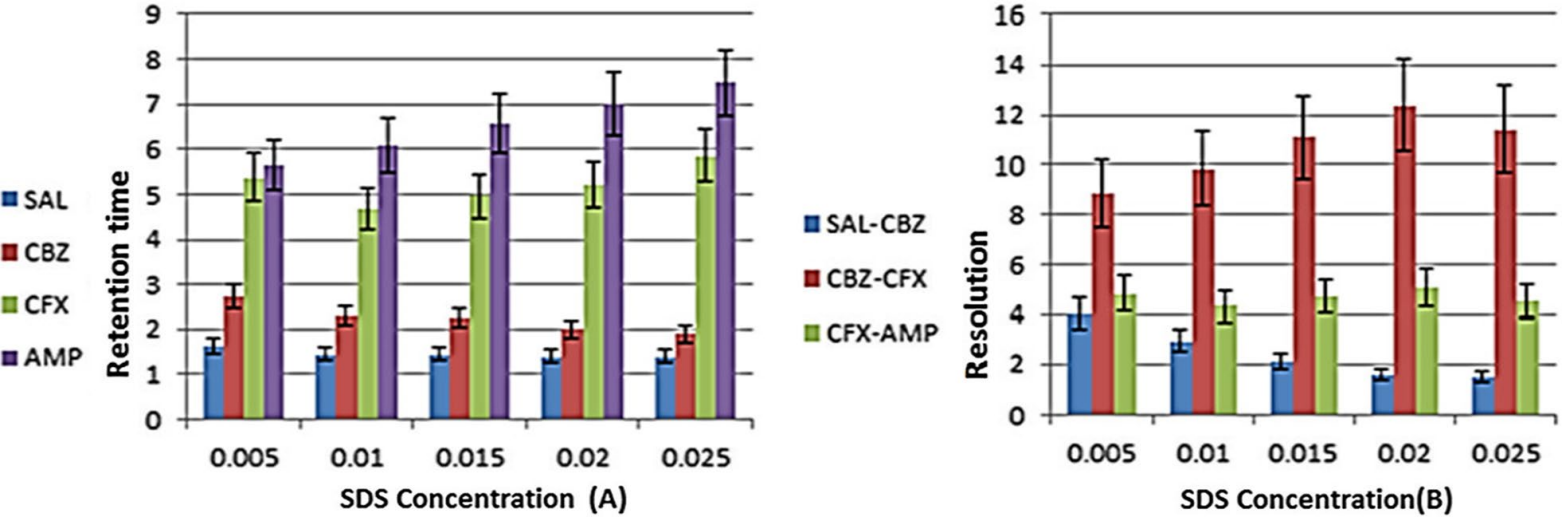
Effect of changing SDS concentration on **A** retention time and **B** resolution of the studied drugs with fixed 0.02 mol/LBrij-35 concentration

When the concentration of one surfactant was changed while the concentration of the other was fixed, the retention time was obviously affected. It was discovered that when the concentration of Brij-35 was fixed while the concentration of SDS was increased, each drug behaved differently in terms of retention time. SLB shows constant retention time, CFP shows decreasing in retention time; CFX shows decreasing in retention time at 0.01 mol/L SDS then increase again at higher SDS concentrations and AMP shows linear increase in retention time. Finally we chose 0.01 mol/L SDS as optimum concentration for separation of the cited drugs as it gives the best separation at lowest retention time. 0.005 mol/L SDS show lowest retention time for separation but the peak for CFX shows splitting i.e. appears as two overlapped peaks. Increasing SDS concentration above 0.01 mol/L led to increase in the retention time for AMP leading to increase in run time, mobile phase consumption and decrease resolution of SLB and CFP gradually as shown in Fig. 1.

**Fig. 2:**
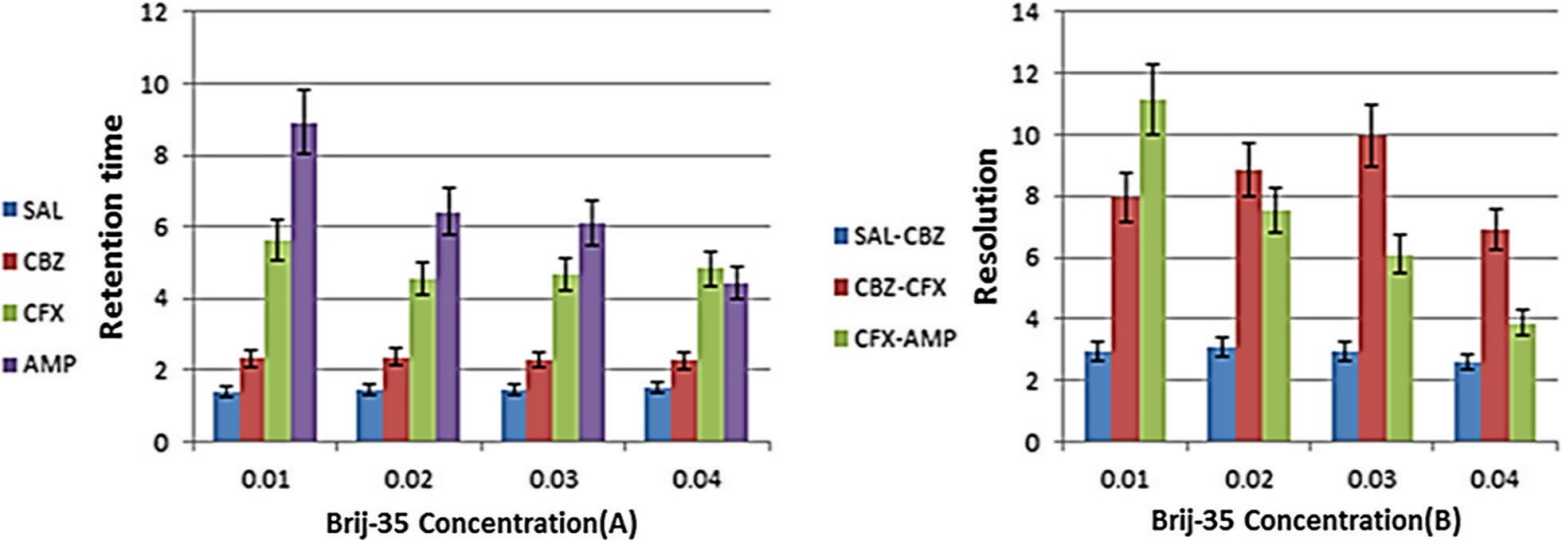
Effect of changing Brij-35 concentration on **A** retention time and **B** resolution of the studied drugs with fixed 0.01 mol/L SDS concentration

On the other hand on fixing SDS concentration at 0.01 mol/L and changing Brij-35 concentration, each drug also shows different behavior in terms of retention time. SLB and CFP show constant retention time, CFX shows decrease in retention time after 0.01 mol/L Brij-35 then slight increase in retention time from 0.02 mol/L to 0.04 mol/L Brij-35 and AMP shows gradual decrease in retention time by increasing Brij-35 concentration. Finally we take 0.03 mol/L Brij-35 as optimum concentration for separation of the studied drug as it show good separation at lowest run time. Increasing concentration of Brij-35 above 0.03 mol/L shows overlap between CFX and AMP as shown in Fig. 2.

**Fig. 3:**
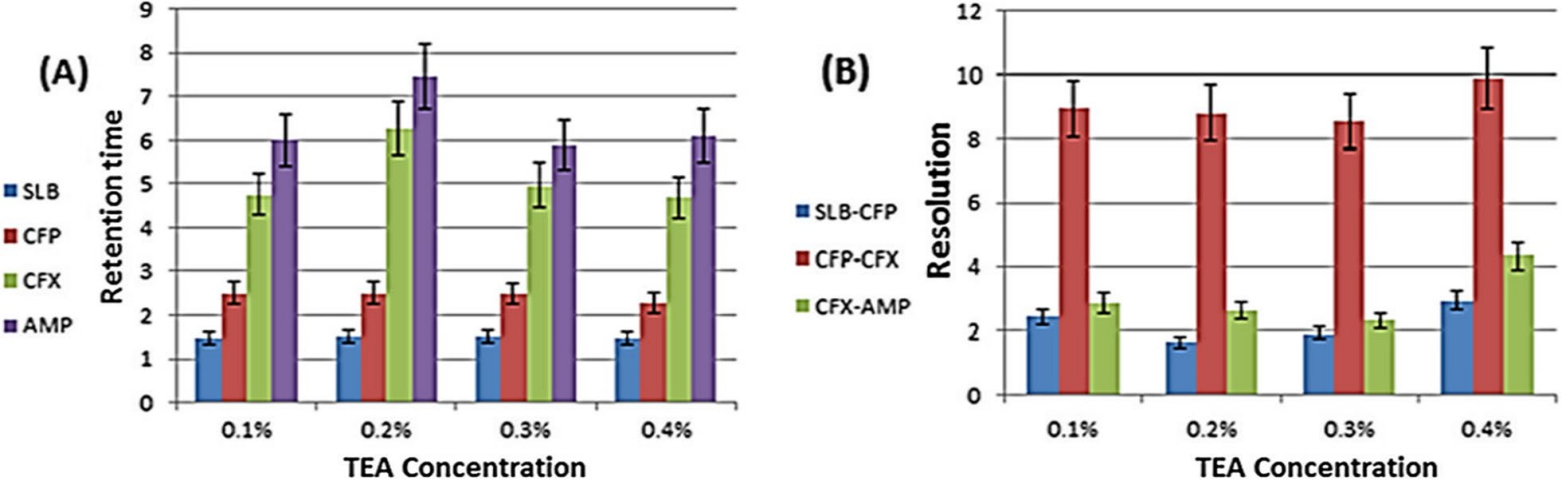
Effect of changing TEA concentration on **A** retention time and **B** resolution of the studied drugs with fixed 0.01 mol/L SDS and 0.03 mol/L Brij-35concentrations

In addition, different percentages of TEA, a quaternary amine compound, were tried as its polarity plays an important role in improving separation of SLB and CFP. Also, it was found that TEA improves peak shape. To know the best used TEA concentration, which gives the optimum separation, we fix the concentration of SDS at 0.01 mol/L and Brij-35 at 0.03 mol/L and study the effect of changing the concentration of TEA on separation of the studied drugs. Each drug shows different behavior where, SLB shows constant retention time over all studied concentrations. CFP shows also constant retention time but slight decrease of retention time at 0.4% TEA concentration. Both CFX and AMP show similar behavior, retention time increase firstly by increasing TEA concentration then decrease after 0.2% TEA concentration as shown in Fig. 3. Finally, we took 0.4% TEA as the optimum concentration for separation as it gives the best separation at lowest run time. Therefore, after studying these factors we use mobile phase consisting of 0.01 mol/L SDS, 0.03 mol/L Brij-35 and 0.4% TEA. pH was adjusted at 2.8 and wavelength at 215 nm. Throughout the study, the stationary phase was regularly cleaned with water and then for 15 min with a mixture of water: MeOH (1:1) mixture. This flushing is crucial to prevent surfactant precipitation and protect the column against salt crystallization. The stationary phase is then regenerated by washing with 100% methanol to remove the surfactant adsorbed.

#### Optimization of stationary phase

Two different columns were tried in the separation process (Isère C_18_ BDS and Vdsphere C18 column), the results showed that Isère C_18_ BDS column give best separation for all studied drugs.

#### Optimization of wavelength

Effect of different wavelengths on detection of drugs was studied by comparing the results of detection at different wavelengths (210, 215, 240 nm) using DAD detector under the same experimental conditions. The results showed that more peak area and height were observed at 215 nm wavelength when compared with other wavelengths.

### Method validation

The goal of validation of an analytical method is to show that it is appropriate for the task at hand. According to the International Conference of Harmonization (ICH) requirements, the created approach was verified [21].

#### Calibration curve and linearity

For the four selected drugs, the linearity ranges that obeys Beer's Law were evaluated, and seven concentrations were chosen over the specified range (10–200 µg mL^−1^), and these concentrations were injected in triplicates. The calibration curves obtained by plotting the peak area versus concentration for each tested drug were subjected to linear regression analysis. High findings of linearity across the required range (R ˃ 0.999) demonstrated excellent linearity. Table 1 shows the results.

**Table 1:** Linear regression and system suitability parameters for the simultaneous determination of the drugs under study using the proposed HPLC method

Parameters	Drug name			
	SLB	CFP	CFX	AMP
Flow rate (mL/min)	1.00	1.00	1.00	1.00
Retention time (min) ± RSD	1.45 ± 0.06	2.29 ± 0.08	4.68 ± 0.06	6.11 ± 0.16
Resolution (Rs) (> 2)*	–	2.95	9.89	4.33
Selectivity factor ( ∝) (≥ 1)*	2.07	2.85	1.94	1.77
Tailing factor (T) (≤ 2)*	1.19	1.12	1.09	1.11
Linearity range (μg/mL)	10–200	10–200	10–200	10–200
Linearity equation	Y = 5446 X + 11,558	Y = 20,459 X − 30,059	Y = 26,631 X − 6047.4	Y = 27,127 X − 29,402
R^2^	0.9998	0.9998	0.9998	0.9999
Accuracy (%R ± RSD) (n = 3)	98.39 ± 1.23	99.97 ± 0.76	100.35 ± 0.66	99.80 ± 1.07
LOD (μg mL^−1^)	2.38	2.01	2.00	2.42
LOQ (μg mL^−1^)	7.20	6.09	6.07	7.34

*System suitability tests reference values

#### Limit of detection and limit of quantification (LOD and LOQ)

LODs and LOQs were calculated as LOD = 3.3 × SD/Slope and LOQ = 10 × SD/Slope, respectively. They expressed the sensitivity of the method. The results were stated in Table 1.

#### Accuracy

How closely the experimental values matches the true value is what is meant by accuracy. It was evaluated by using mean percentage recovery of QCH, QCM and QCL measured in triplicate injections. The results stated in Table 1 confirmed trueness of the proposed method.

#### Precision

Precision refers to how close the measurements are to each other. By injecting samples three times on the same day and three consecutive days, inter-day and intra-day results were stated to evaluate the precision of the studied method. As shown in Table 2, the results show excellent precision by variation coefficient less than 2%.

**Table 2:** Intra-day and inter-day precision for determination of the drugs under study

Drug	QC sample (μg mL^−1^)	Inter-day	Intraday
		(CV)*	(CV)*
SLB	150	0.4	0.7
	100	0.4	1.7
	50	0.08	0.08
CFP	150	0.6	0.6
	100	0.8	0.8
	50	0.1	0.1
CFX	150	0.8	0.1
	100	0.2	0.3
	50	0.9	0.3
AMP	150	0.7	0.3
	100	0.9	0.5
	50	0.1	0.7

*Coefficient of variation (%) = (SD/mean) × 100

#### Specificity and selectivity

**Fig. 4:**
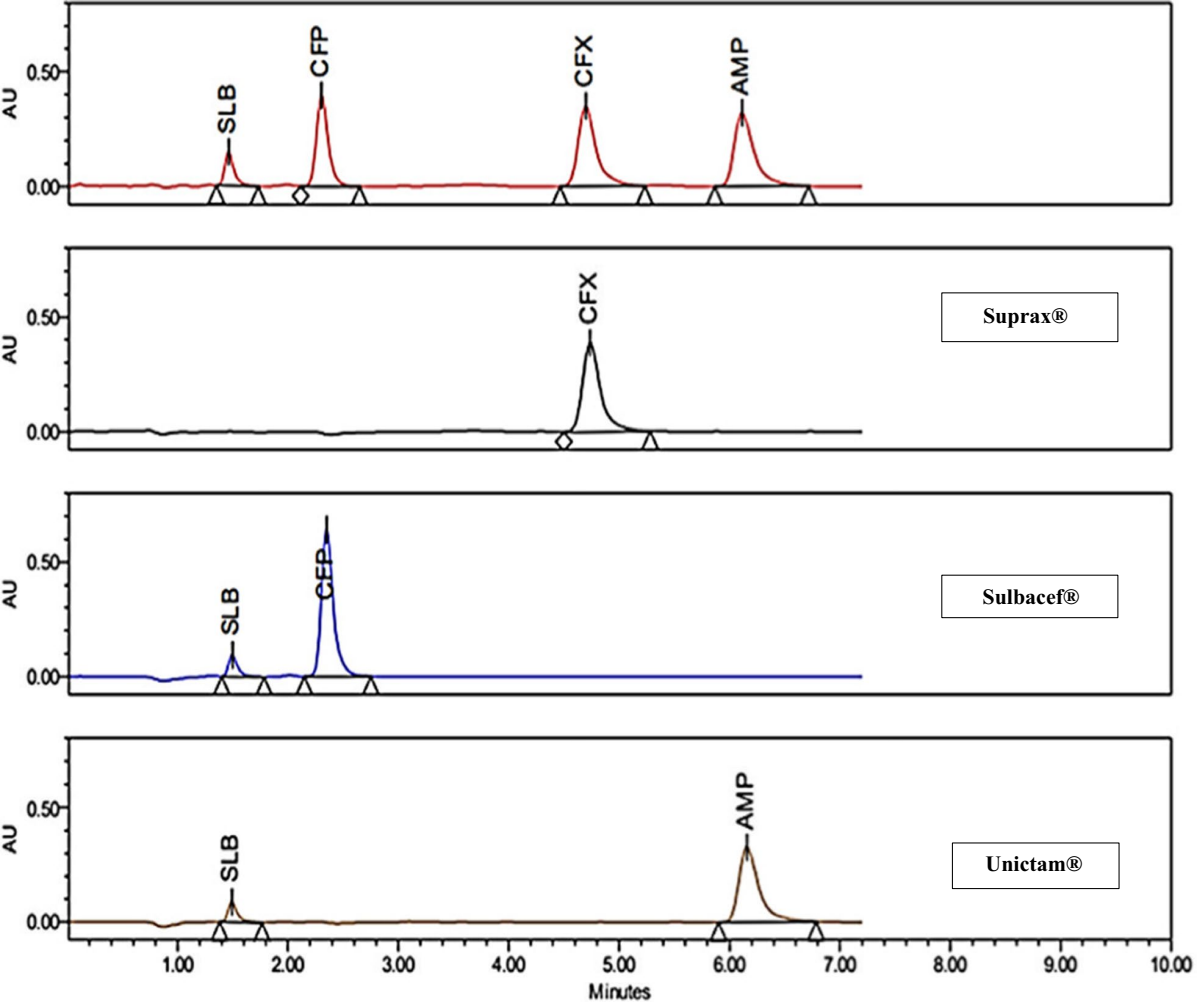
HPLC chromatogram showing separation of the drugs under study in their laboratory prepared mixture and pharmaceutical dosage forms. Mobile phase: 0.03 mol/L Brij-35, 0.01 mol/L SDS and 0.4% TEA at pH 2.8 and flow rate 1 mL/min

The capacity of an analytical procedure to determine the examined drugs in the presence of interferences is known as selectivity. Each sample and its standard solutions had identical chromatograms. The drugs under study were clearly resolved without any interference from excipients found in any dosage form used as shown from good percentage recoveries in Table 1. Figure 4 shows a representative chromatogram of the quality control solution (QC) to show that there are no peaks from various additives and excipients.

#### Robustness

The robustness of the method was investigated by making minor changes in chromatographic conditions such as flow rate (1.00, 0.95, 1.05), column temperature (40, 38, 42 °C), and wavelength (215, 214, 216 nm) to demonstrate constant peak area and retention time. As a result, the resolution between peaks and the recovery percentage were unaffected. Additional file 1: Table S2 shows the results.

#### System suitability parameters

To make sure the system is operationally sound, system suitability parameters are assessed prior to commencing HPLC analysis. Calculating these parameters enabled it to be done (selectivity, resolution, capacity factor, and column efficiency and height equivalent theoretical plates). All of them are inside the reference range, according to the results, as shown in Table 1, ensuring the system's suitability.

### Analytical application

Drugs under study are present in the market mainly in vial dosage forms and also other forms like tablet, capsules and suspensions. They may present either alone or in combinations except SLB always present in combinations. The method under study was applied for the determination of the four antibiotics in three different dosage forms, two vial dosage forms and one capsule dosage form. As SLB present in a combination dosage form with CFX in Indian market and other markets but not in the Egyptian market, so we made the application on CFX alone, the proposed method present a good separation for SLB and CFX, so we expect a good separation if we applicate the proposed method on the combination dosage form. No interferences were found from excipients in any of the applied dosage forms. The obtained data stated in Table 3, show that the studied method is excellent for separation of the four antibiotics under study in combinations of each other or in single component dosage form.

**Table 3:** Results obtained for analysis of different dosage forms containing the drugs under study

Dosage form	Composition	Labeled content (per table)	Assayed content	Found% ± RSD
Unictam® Vial	AMP	1000 mg AMP + 500 mg SLB	150 mg AMP + 75 mg SLB	102.25 ± 0.22
	SLB		100 mg AMP + 50 mg SLB	101.68 ± 0.18
			50 mg AMP + 25 mg SLB	100.28 ± 0.37
Sulbacef® Vial	CFP	1000 mg CFP + 500 mg SLB	150 mg CFP + 75 mg SLB	100.72 ± 0.07
	SLB		100 mg CFP + 50 mg SLB	100.96 ± 1.88
			50 mg CFP + 25 mg SLB	100.16 ± 0.14
Suprax® Capsules			150 mg	100.29 ± 0.23
	CFX	200 mg	100 mg	100.93 ± 0.03
			50 mg	100.86 ± 0.33

### Assessing the greenness of the proposed method

Recently, there is a trend toward green chemistry in all methods of analysis, spectrophotometry or chromatography or electrochemistry…et al., for example micellar HPLC method for separation of anti-diabetic drugs [22], anti-hypertensive drugs [23] and drugs for treatment of common cold [24].

As discussed in the introduction, drugs under study were separated using several HPLC methods but all reported methods were using mobile phases consisting of organic solvents in different proportions. The proposed method is totally free from organic solvents so it's regarded a green analytical method. The percentage of organic solvent in any analytical method is considered a key principle in assessing the method's greenness; thus, decreasing the percentage of organic solvents in the mobile phase increases the method's greenness. The method's greenness was assessed using a new tool, the Analytical Greenness Metric (AGREE) [25], which was founded on the twelve principles of green analytical chemistry (GAC). AGREE presents a clock-shaped graph divided into 12 sections, each representing a different GAC principle. AGREE's color code ranges from red to yellow to green. The color red represents high impact, the color yellow represents medium impact, and the color green represents low impact. A score was displayed in the center of the assessment shape to indicate the overall greenness of the method.

The proposed method compared with four reported methods mentioned in introduction [14, 18–20]. Two of them are HPLC methods differ in the analyzed drugs and mobile phase composition. The other two methods are a micellar electrokinetic chromatographic method and a HPTLC method. As shown in Table 4 the proposed method has eight green, three yellow and only one red part so it has the lowest impact on environment compared with the other methods which contain more red and yellow parts. So the method is superior in greenness regarding to other methods.

**Table 4:** Comparison between the proposed and reported MLC methods for determination of drugs under study

Method	Proposed method	Reported method [14]	Reported method [18]	Reported method [19]	Reported method [20]
Technique	MLC, C_18_, HPLC–UV	Bonded b-CD column, HPLC–UV	HPLC–UV Hyper ODS2, Column C_18_	HPTLC	Micellar electrokinetic capillary electrophoretic
Organic modifier	Totally free	Tetraethylammonium acetate	Mixture of 45 mL Acetonitrile and 55 mL of water	Acetone‒ethanol‒ethyl acetate‒2% sodium dodecyl sulfate‒glacial acetic acid (3:2:4:1:0.5, V/V)	0.02 M monobasic sodium phosphate Adjusted to pH 3.0 (with 40% phosphoric acid) and acetonitrile)
Analytes similarity	AMP, SLB CFP, CFX	AMP, CFP, SLB	CFX, SLB	CFP, SLB	AMP, SLB
Runtime	6 min	28.3 min	3.6	5 min	20 min
GAPI assessment					
AGREE					

Other new assessment tool used for greenness assessment to evaluate each item involved in chromatographic procedures is the GAPI tool [26]. Each of the 5 pentagrams that make up GAPI represents a stage of the analytical process, such as sample collection, preservation, transit, storage, and preparation. They also cover the usage of instruments, waste, waste treatment, solvents, and reagents. Red denotes a high ecological impact, yellow, a low ecological impact, and green, the most environmentally friendly color, according to the GAPI color code. The proposed method compared with the same reported methods in the AGREE assessment tool as shown in Table 4. GAPI pentagram for the proposed method has the least number of red zones (only two) comparing to all the compared methods except the compared HPTLC method which has also two red zones but, our method has more yellow zones than the compared HPTLC method so it's also superior to the compared HPTLC method. The two red zones in our method indicate the mandatory offline sampling and the placement of analytical devices within quality control laboratories far from the production sites in all pharmaceutical factories. Nine green pentagram in GAPI tool with eight green part in AGREE assessment tool proves the proposed method superiority.

## Conclusion

The creation of chemical procedures that lessen or do away with the use of hazardous materials is known as "green chemistry." Green analytical chemistry is required for human health, cleaner air, less hazardous chemical release into the atmosphere. The four drugs under study were separated by various HPLC methods but all of them involving the use of organic solvent in the composition of mobile phase in different proportions, but in our method, no organic solvents were used at all, this is what we want to achieve from our research. The use of organic solvents has a significant environmental impact because their production and disposal pose economic and biohazards issues. MLC is a great alternative to traditional HPLC, which uses an organic solvent as the primary component of the mobile phase. The use of SDS in conjunction with Brij-35 is being investigated as an alternative to the presence of organic solvents. The chromatographic performance of mixed micellar mobile phase for separation of cited drugs was investigated in this study, and the method was applied to marketed pharmaceutical dosage forms.

### Supplementary Information

**Additional file 1: Table S1. **Chemical structures of the studied drugs. **Table S2. ** Robustness of the mentioned method applied on concentration of 150 μg mL-1 for all drugs.

## Data Availability

All data generated or analyzed during this study are included in this published article.

## Abbreviations

CFP: Cefoperazone
SLB: Sulbactam
CFX: Cefixime trihydrate
AMP: Ampicillin
MLC: Micellar liquid chromatography
SDS: Sodium dodecyl sulphate
Brij-35: Polyoxyethylene-23- lauryl ether
TEA: Tri-ethylamine
ICH: International Conference on Harmonization
HPLC: High performance liquid chromatography
GAPI: Green Analytical Procedure Index
AGREE: Analytical Greenness metric approach
