# Supplementary material for: Development of green micellar HPLC–DAD method for simultaneous determination of some sulbactam combinations used in COVID-19 regimen
Source: BMC Chem. 2023 Aug 5;17(1):94. doi: 10.1186/s13065-023-01006-0 (PMC10403863; doi:10.1186/s13065-023-01006-0)
Supplement: Supplementary file 1 — Additional file 1: Table S1. Chemical structures of the studied drugs. Table S2. Robustness of the mentioned method applied on concentration of 150 μg mL-1 for all drugs. [file 13065_2023_1006_MOESM1_ESM.docx]

**Table S1.** Chemical structures of the studied drugs

| **Drug** | **Chemical structure** |
| --- | --- |
| SLB | 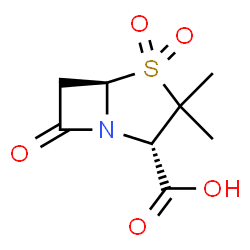 |
| CFP | 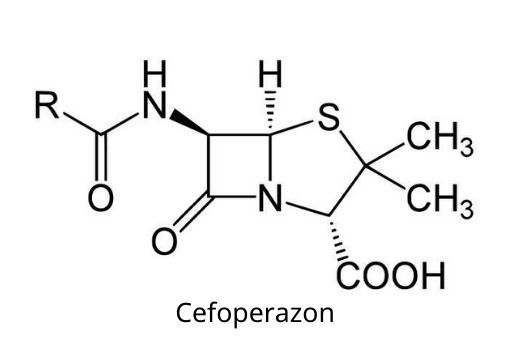 |
| CFX | 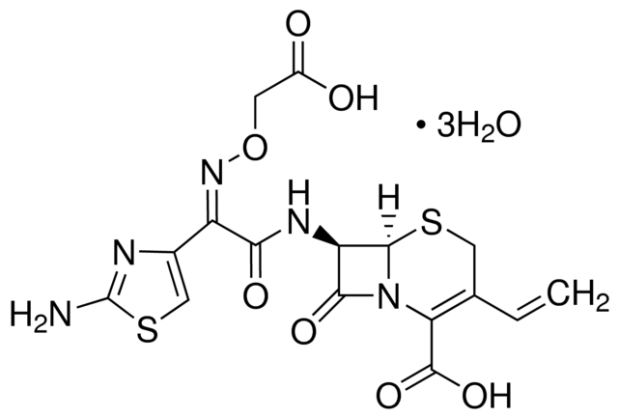 |
| AMP | 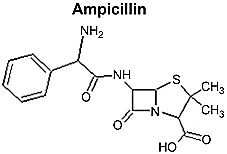 |

**Table S2.** Robustness of the mentioned method applied on concentration of 150 μg mL^-1^ for all drugs.

| **Retention time** | | | | **Peak Area** | | | | **Parameter affected** |
| --- | --- | --- | --- | --- | --- | --- | --- | --- |
| **AMP** | **CFX** | **CFP** | **SLB** | **AMP** | **CFX** | **CFP** | **SLB** |  |
|  |  |  |  |  |  |  |  | **Temperature ( ̊C)** |
| 5.76 | 4.90 | 2.33 | 1.48 | 3947272 | 3947272 | 3061745 | 921602 | 38 |
| 5.91 | 4.63 | 2.29 | 1.46 | 3959929 | 3932974 | 3047223 | 909496 | 40 |
| 6.31 | 4.45 | 2.25 | 1.44 | 3959488 | 3957285 | 3043135 | 909196 | 42 |
| 4.72 | 4.85 | 1.61 | 1.61 | 0.18 | 0.30 | 0.32 | 0.78 | **RSD%** |
|  |  |  |  |  |  |  |  | **Flow rate (mL/min)** |
| 5.73 | 4.75 | 2.39 | 1.45 | 3820787 | 3943738 | 3211640 | 936178 | 0.95 |
| 5.98 | 4.63 | 2.29 | 1.46 | 3939929 | 3932974 | 3097223 | 909196 | 1 |
| 6.26 | 4.84 | 2.19 | 1.45 | 3808106 | 3867849 | 3137740 | 862825 | 1.05 |
| 4.36 | 2.17 | 4.36 | 0.40 | 1.88 | 1.18 | 1.86 | 4.10 | **RSD%** |
|  |  |  |  |  |  |  |  | **Wavelength (nm)** |
| 5.98 | 4.63 | 2.29 | 1.46 | 3959929 | 3932974 | 3047223 | 892543 | 214 |
| 5.98 | 4.63 | 2.29 | 1.46 | 3959929 | 3932974 | 3047223 | 909196 | 215 |
| 5.98 | 4.63 | 2.29 | 1.46 | 4027571 | 3974949 | 3078921 | 909196 | 216 |
| 0 | 0 | 0 | 0 | 0.98 | 0.61 | 0.59 | 1.06 | **RSD%** |
